# Patient with neuromyelitis optica and inflammatory demyelinating lesions comprising whole spinal cord from C2 level till conus: case report

**DOI:** 10.1186/1471-2377-9-56

**Published:** 2009-10-23

**Authors:** Zeljka Petelin Gadze, Sanja Hajnsek, Silvio Basic, Davor Sporis, Goran Pavlisa, Sibila Nankovic

**Affiliations:** 1 Department of Neurology of the School of Medicine and Zagreb University Hospital Centre, Zagreb, Croatia; 2 Department of Neurology, Dubrava University Hospital, Zagreb, Croatia; 3 Department of Radiology of the School of Medicine and Zagreb University Hospital Centre, Zagreb, Croatia

## Abstract

**Background:**

Neuromyelitis optica (NMO) is an idiopathic, severe, inflammatory demyelinating disease of the central nervous system, that causes severe optic neuritis and myelitis attacks. Early discrimination between multiple sclerosis (MS) and NMO is important, as optimum treatment for both diseases may differ considerably.

**Case Presentation:**

We report a case of a patient who initially presented as longitudinally extensive transverse myelitis (LETM), having spastic upper extremities diparesis and spastic paraplegia, C2/C3 sensory level and urinary incontinence, as well as extensive inflammatory spinal cord lesions from C2 level to conus. After 5 months the patient had another attack of transverse myelitis, had electrophysiological findings consistent with optic neuritis, was seropositive for NMO-IgG (aquaporin-4 IgG) and thus fulfilled NMO diagnostic criteria. Following treatment of disease attacks with pulse corticosteroid therapy and intravenous immunoglobulins, we included oral azathioprine in a combination with oral prednisone in the therapy. Since there was no significant clinical improvement, we decided to use cyclophosphamide therapy, which resulted in good clinical improvement and gradual decrease of cord swelling.

**Conclusion:**

In this NMO case report we wanted to emphasize the extensiveness of inflammatory spinal cord changes in our patient, from C2 level to conus. In the conclusion it is important to say that accurate, early diagnosis and distinction from MS is critical to facilitate initiation of immunosuppressive therapy for attack prevention.

## Background

Neuromyelitis optica (NMO) or Devic's syndrome is an idiopathic, severe, inflammatory demyelinating disease of the central nervous system that selectively affects optic nerves and spinal cord. It tends to spare the brain early in the disease course. Early discrimination between multiple sclerosis (MS) and NMO is important, as optimum treatment for both diseases may differ considerably. In contrast to typical MS, clinical experience and case series suggest that NMO requires long-term immunosuppressive therapy. Diagnostic criteria for NMO from 1999 have been revised in 2006 by Wingerchuk et al. Diagnosis requires absolute criteria and at least two of three supportive criteria. Absolute criteria include presence of optic neuritis and acute myelitis, and supportive criteria negative brain magnetic resonance imaging (MRI) at disease onset, spinal cord MRI with contiguous T2-weighted signal abnormality based centrally in the cord and extending over 3 or more vertebral segments (LETM - longitudinally extensive transverse myelitis), and NMO-IgG (aquaporin-4 IgG) seropositive status [1,2]. NMO-IgG has facilitated an appreciation that the spectrum of NMO is wider than previously recognized, and includes patients with recurrent longitudinally extensive transverse myelitis, recurrent isolated optic neuritis, and Japanese opticospinal MS [3]. A variety of encephalopathic presentations may occasionally be encountered in NMO-IgG seropositive patients, most commonly in children [4].

In this article we report a case of a patient who initially presented as LETM, having spastic upper extremities diparesis and spastic paraplegia, C2/C3 sensory level and urinary incontinence, as well as extensive inflammatory spinal cord lesions from C2 level to conus. After 5 months LETM evolved to neuromyelitis optica.

## Case Presentation

Patient A.V., Caucasian, born in 1966, with a history of rhematic fever at the age of 8, in May 2006 was operated in another institution due to left paramedian L5-S1 disc herniation, which clinically presented with plegia of the left leg, pain and hypoesthesia in the left L5 - S1 dermatome, and urinary incontinence. After operation and physical rehabilitation her neurological status improved. However, at the beginning of August 2006, motor weakness of the left leg worsened, and was accompanied by development of weakness of the right leg. In a few days she became paraplegic with Th4 sensory level and urinary incontinence. MRI of the thoracal spinal cord revealed edematous enlargement extending from Th4 to Th6 level, suspect of expansive process, but during second operation 5 cm long arachnoid cyst was revealed and neurosurgeons performed fenestration of the cyst. Soon after she was sent to physical rehabilitation. In the next few months her condition slightly improved, she again began to walk, with assistance (crutches). In December 2006 her neurological status again started to deteriorate, in the next few months weakness of legs worsened, and she also developed weakness of both arms, accompanied with paresthesiae and dysesthesiae in arms and legs.

**Figure 1:**
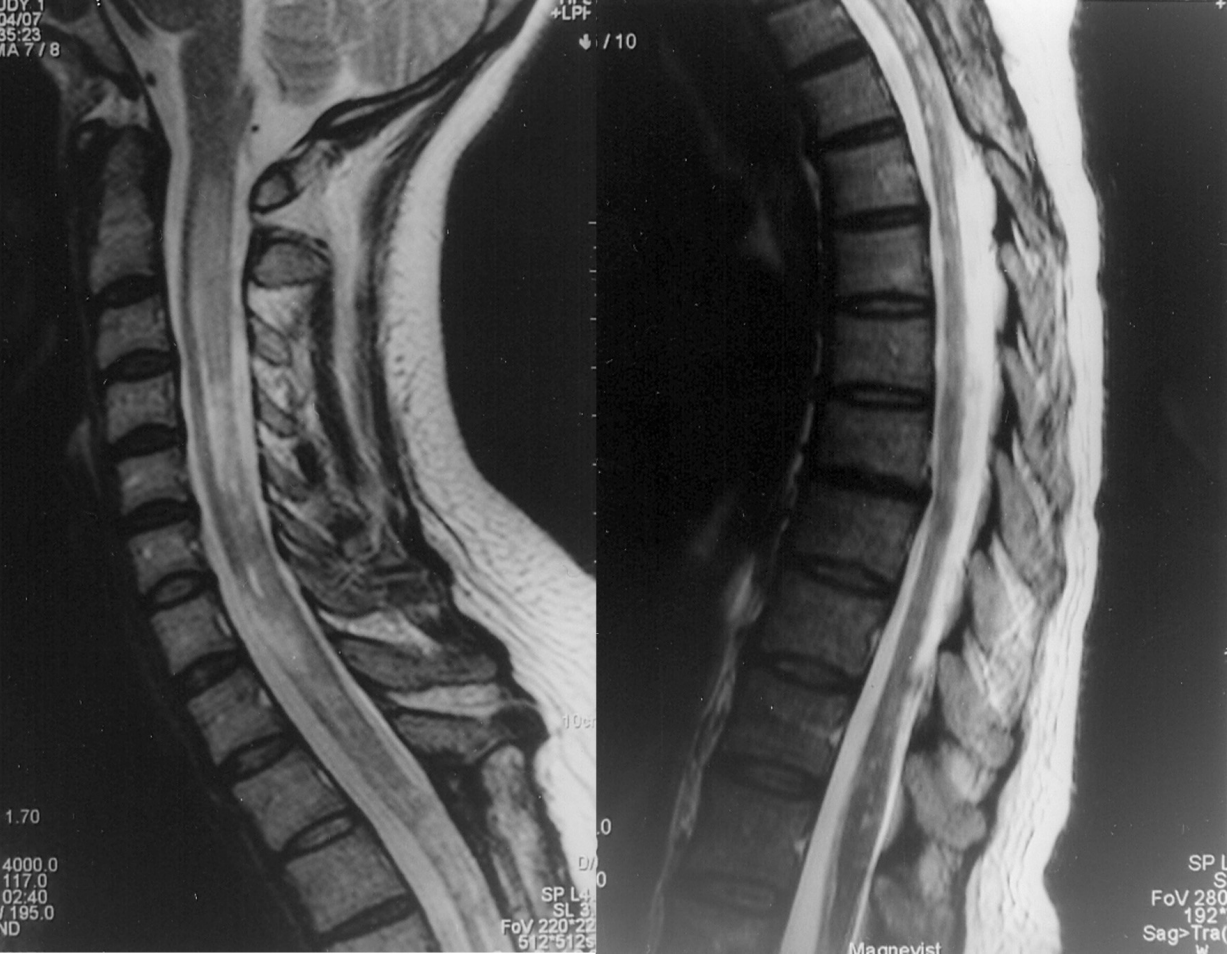
**Spinal cord MRI (T2W images) performed in April 2007**. MRI shows extensive T2 hyperintense changes along the spinal cord from C2 level till conus. Those changes were most prominent in the cervical and cervicothoracal spinal cord, with widening of central canal in some places and edematous enlargements, as well as with zones of necrosis.

**Figure 2:**
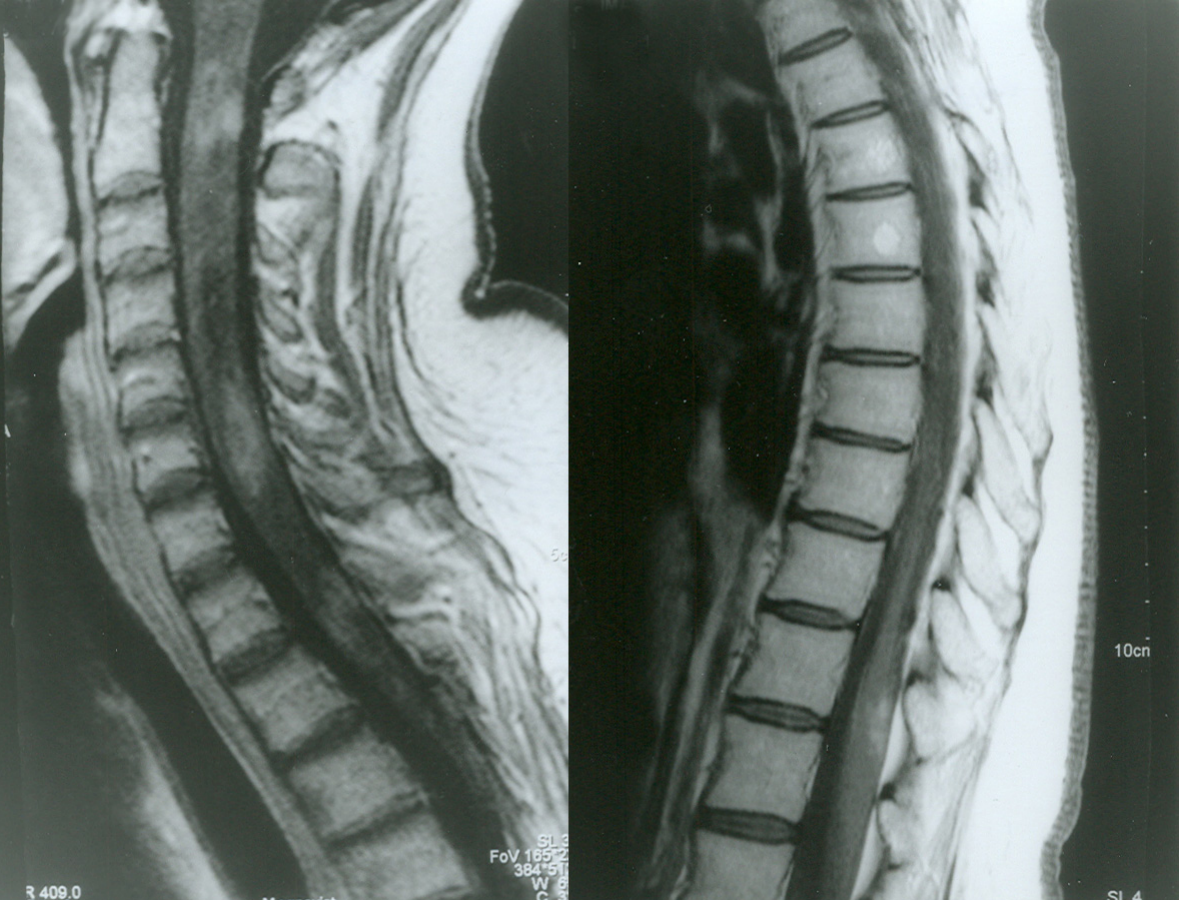
**Spinal cord MRI (T1W images with gadolinium) performed in April 2007**. MRI shows few areas of pathological gadolinium inhomogenous and ring enhancement, at C2, C4-C5 and Th1 levels. Those changes pointed to aggressive inflammatory changes.

In April 2007 she was admitted to the Department of Neurology of the University Hospital Centre Zagreb. At that time she had spastic upper extremities diparesis, more pronounced in the left arm, and spastic paraplegia, with brisk deep tendon reflexes and bilateral positive Babinski sign, as well as C2/C3 sensory level and urinary incontinence. MRI revealed T2 hyperintense changes along the spinal cord from C2 level till conus, with zones of necrosis, most prominent in the cervical and cervicothoracal spinal cord, with widening of central canal in some places and edematous enlargements. There were few areas of pathological Gadolinium inhomogenous and ring enhancement, at C2, C4-C5 and Th1 levels. Those changes pointed to aggressive inflammatory changes (Figure 1 and 2, April 2007). Brain MRI was normal. In cerebrospinal fluid (CSF) there were 10 cells/mL (5 lymphocytes and 5 neutrophils), raised total protein level (0.65 g/L), and positive oligoclonal bands (OCB). Immunological tests were normal as well as tumor markers, paraneoplastic antibodies and analysis of serum and CSF for the possible presence of infectious causes (Borrelia burgdorferi, neurotropic viruses including HIV, Mycobacterium tuberculosis, Treponema pallidum, Mycoplasma pneumoniae). Visual evoked potentials (VEP) finding was also normal. We made the diagnosis longitudinal extensive transverse myelitis (LETM) and started treatment with intravenous methylprednisolone in a dose of 1000 mg/day for 5 days, followed by intravenous immunoglobulins (IVIG) in a dose of 400 mg/kg/day during 5 days, and physical therapy. Slight improvement of her symptoms and regression of weakness in upper extremities was achieved. Baclofen and diazepam were also included in the therapy. Control MRI performed after one month showed decrease of edematous enlargement of the spinal cord. She was sent to rehabilitation.

After 5 months she again progressively developed weakness, paresthesiae and dysesthesiae in arms, more pronounced in the left arm, as well as transitory blurring of vision on both eyes. Control MRI of the spinal cord revealed progression of extensive demyelinating lesions in the cervical segment. Repeated brain MRI was normal. Control VEP revealed conduction disturbances (prolonged latencies) in both optic pathways. In the meantime, we have received positive finding of NMO-IgG antibodies from Mayo Medical Laboratories. According to that finding, clinical presentation and MRI finding we made the diagnosis neuromyelitis optica. Patient was again treated with intravenous methylprednisolone and IVIGs, that resulted in minimal clinical improvement of weakness in upper extremities, but there was no improvement of spastica paraplegia. She remained restricted to bed. In the following months we also included oral azathioprine in a combination with oral prednisone in the therapy, but since there was no significant clinical improvement, we considered other treatment modalities. In January 2008 we started treatment with intravenous cyclophosphamide (1 g/m^2^) in a combination with methylprednisolone (1000 mg), once a month during six months. Partial remission of disease activity has been achieved. At the moment patient can use both her arms and can stand supported by sticks. Her recent MRI of the spinal cord revealed gradual decrease of cord swelling and T2 signal hyperintensity. In the meantime, following steroid therapy, she developed diabetes mellitus and osteoporosis, that are being treated successfully.

## Consent

Written informed consent was obtained from the patient for publication of this case report and accompanying images. A copy of the written consent is available for review by the Editor-in-Chief of this journal.

## Discussion

Neuromyelitis optica (NMO) was first identified in the 19^th ^century, as a monophasic, destructive disorder affecting the spinal cord and both optic nerves, but sparing the remainder of the central nervous system (CNS). The position of NMO within the group of CNS demyelinating diseases has been long debated - whether this clinical entity is a severe variant of MS, a form of acute disseminated encephalomyelitis, or a distinct disease [5,6]. It has gradually become accepted that the spectrum of idiopathic NMO is broader than suggested by the historical definition. The recent discovery of serum autoantibody marker NMO-IgG in 2004 further advances the hypothesis that NMO is actually a distinct disease [3]. NMO-IgG is 73% sensitive and 91% specific for distinguishing NMO from optic-spinal presentations of classical MS. The target antigen of NMO-IgG is aquaporin 4 (AQP4), glial water channel protein, that facilitates water transport, especially in "stress situations" such as brain injury. It is component of the dystroglycan protein complex located in astrocytic foot processes at the blood-brain barrier. Data suggest that autoantibodies to aquaporin 4, derived from peripheral B cells, cause the activation of complement, inflammatory demyelination, and necrosis that is seen in neuromyelitis optica [7,8]. It is also important to emphasize that patients presenting with a first-ever LETM event, who are found to be NMO-IgG seropositive, have a 56% risk of LETM recurrence or optic neuritis (conversion to NMO) during the subsequent 12 months [9]. With the positive NMO-IgG finding we confirmed the diagnosis of NMO in our patient, who satisfied all absolute and supportive diagnostic criteria [1].

According to the data from the literature, most patients with NMO, perhaps more than 90%, have relapsing rather than monophasic disease [9], which was also the case in our patient, but was not recognized as that in the beginning of the disease. Our patient met the features of relapsing NMO, such as female dominance, older age at onset, and probably autoimmune disease - we could assume that steroid therapy unmasked autoimmune diabetes mellitus [10].

For many neurologists, the current standard preventive approach for NMO includes oral azathioprine in a combination with oral prednisone, rituximab (chimeric anti-CD20 monoclonal antibody), mitoxantrone, IVIGs, and cyclophosphamide [11-17]. The best evidence (although all from retrospective series) for long-term immunosuppression in NMO is with azathioprine and rituximab, that are considered first line therapies, while cyclophosphamide is generally considered as a second line agent in NMO. In a recently published retrospective multicenter case series of NMO patients treated with rituximab, that included 25 patients (including 2 children), 23 of whom experienced relapses despite use of other drugs before rituximab, treatment with rituximab appeared to reduce the frequency of attacks, with subsequent stabilization or improvement in disability.[14] Jarius et al reported that treatment with immunosuppressants such as rituximab, azathioprine and cyclophosphamide resulted in a marked reduction in antibody levels as well as in relapse rates, demonstrating a strong relationship between AQP4-Abs and clinical state. Cyclophosphamide resulted in a long-lasting relapse-free interval in one of their patients, with 10 relapses within 1295 days (2.82/year) prior to initiation of therapy but only one within 1610 days (0.23/year) under therapy [15]. Immunoablative cyclophosphamide was also successful in halting relapses in a patient with systemic lupus erythematosus-associated NMO who was unresponsive to high-dose oral and intravenous corticosteroids, intravenous immunoglobulin, mycophenolate mofetil, tacrolimus, low-dose daily oral cyclophosphamide and rituximab [17].

After treating diseases attacks with pulse corticosteroid therapy and IVIGs in our patient, we included oral azathioprine in a combination with oral prednisone in the therapy, but since there was no significant therapeutic response, we decided to use cyclophosphamide therapy. That resulted in good clinical improvement and gradual decrease in cord swelling and T2 signal hyperintensity. We were also considering to use rituximab, but at that time this agent was not approved for treating NMO in Croatia.

It is important to point out that one does not need to wait for a second attack of transverse myelitis or optic neuritis prior to commencing immunosuppression if NMO-IgG is positive, since NMO-IgG itself predicts a relapsing course. Unfortunately, at the time we received positive finding of NMO-IgG antibodies from Mayo Clinic, our patient experienced relapse. If immunosuppresive therapy had been started earlier in the course of the disease (patient's clinical worsening started to happen one year before admission to our institution), maybe the prognosis for this patient could be more favourable.

## Conclusion

In this case report we wanted to emphasize the extensiveness of inflammatory spinal cord changes in our patient, from C2 level to conus, which was initially attributed to LETM, but after some time evolved to neuromyelitis optica. In the conclusion it is important to say that accurate, early diagnosis and distinction from multiple sclerosis is critical to facilitate initiation of immunosuppressive therapy for attack prevention.

## Competing interests

The authors declare that they have no competing interests.

## Authors' contributions

All authors stated above made substantive intellectual contributions to a published case report. ZPG treated the patient and wrote the article; SH treated the patient, organized the sending of blood samples to Mayo Medical Laboratories for the analysis of NMO-IgG antibodies and helped to draft the manuscript; SB participated in the treatment of patient and helped to draft the manuscript; DS helped in making the diagnosis of the patient; GP helped in making the diagnosis of the patient by performing and analysing brain and spinal cord MR images; SN helped to draft the manuscript.

All authors read and approved the final manuscript.

## Pre-publication history

The pre-publication history for this paper can be accessed here:


